# Metabolic Alterations in Obstructive Jaundice: Effect of Duration of Jaundice and Bile-Duct Decompression

**DOI:** 10.1155/1991/27457

**Published:** 1991

**Authors:** R. N. Younes, N. A. Vydelingum, P. Derooij, F. Scognamiglio, L. Andrade, M. C. Posner, M. F. Brennan

**Affiliations:** 1 The Department of Surgery Memorial Sloan-Kettering Cancer Center New York NY 10021 USA; 2 Memorial Sloan-Kettering Cancer Center 1275 York Avenue New York NY 10021 USA

## Abstract

We examined the effect of prolonged bile duct obstruction, and subsequent biliary decompression, on biochemical and metabolic parameters, using a reversible jaundice model in male Fischer 344 rats. The animals were studied after biliary obstruction for varying periods (4 days, one week, and two weeks) and following decompression. They were sacrificed one or two weeks following decompression. All the rats were compared to sham operated, pair-fed, controls. Obstructive jaundice rapidly increased bilirubin, liver enzymes, serum free fatty acid, and triglyceride levels. Glucose levels were significantly decreased in the jaundice rats compared to their pair-fed controls. Only after two weeks of jaundice was significant hypoalbuminemia observed. Following decompression, all biochemical and metabolic values gradually returned to normal levels, except for albumin. Hypoalbuminemia was not reversed within the two-week post-decompression period. The rats jaundiced for two weeks had significantly higher mortality, compared to the other groups. We conclude that prolonged jaundice adversely affects the metabolic capacity of the rats, with albumin concentration being markedly decreased, and that biliary decompression could not reverse completely all the alterations seen with cholestasis, especially following two weeks of bile duct obstruction.


*HPB Surgery*, 1991, Vol. 5, pp. 35–48
Reprints available directly from the publisher
Photocopying permitted by license only

© 1991 Harwood Academic Publishers GmbH
Printed in the United Kingdom

### METABOLIC ALTERATIONS IN OBSTRUCTIVE JAUNDICE: EFFECT OF DURATION OF JAUNDICE AND BILE-DUCT DECOMPRESSION

R.N. YOUNES, N.A. VYDELINGUM, P. DEROOIJ, F. SCOGNAMIGLIO, L. ANDRADE, M.C. POSNER and M.F. BRENNAN

*The Department of Surgery, Memorial Sloan-Kettering Cancer Center, New York, NY, 10021, USA*

*(Received 15 March 1991)*

We examined the effect of prolonged bile duct obstruction, and subsequent biliary decompression, on biochemical and metabolic parameters, using a reversible jaundice model in male Fischer 344 rats. The animals were studied after biliary obstruction for varying periods (4 days, one week, and two weeks) and following decompression. They were sacrificed one or two weeks following decompression. All the rats were compared to sham operated, pair-fed, controls. Obstructive jaundice rapidly increased bilirubin, liver enzymes, serum free fatty acid, and triglyceride levels. Glucose levels were significantly decreased in the jaundice rats compared to their pair-fed controls. Only after two weeks of jaundice was significant hypoalbuminemia observed. Following decompression, all biochemical and metabolic values gradually returned to normal levels, except for albumin. Hypoalbuminemia was not reversed within the two-week post-decompression period. The rats jaundiced for two weeks had significantly higher mortality, compared to the other groups. We conclude that prolonged jaundice adversely affects the metabolic capacity of the rats, with albumin concentration being markedly decreased, and that biliary decompression could not reverse completely all the alterations seen with cholestasis, especially following two weeks of bile duct obstruction.

KEY WORDS: Jaundice, metabolism, lipids, liver functions, rats

### INTRODUCTION

Obstructive jaundice is associated with high morbidity and mortality in patients submitted to surgical intervention. The timing of bile-duct decompression in relation to the definitive operative procedure is still controversial, with some authors advocating preoperative decompression[1], while others support a concomitant decompression and definitive operation[2]. Several experimental studies have been attempted to elucidate the pathophysiology of extrahepatic bile-duct obstruction, as well as the effects of bile duct decompression on various metabolic parameters[3]. The models usually used by these authors are complex, with extensive operative procedures that further affect organ function. Recently in our laboratory, Posner *et al.*[4] designed a rat model for reversible obstructive jaundice which is simple, reproducible and performed with minor surgical trauma. They studied this model within a four day period, showing that the rats consistently developed jaundice. They were able to reverse the jaundiced condition after four days of

Address correspondence to: Nadarajen A. Vydelingum, Ph.D. Memorial Sloan-Kettering Cancer Center, 1275 York Avenue, New York, NY 10021, USA

decompression. In the present study, we evaluated the effect of prolonged jaundice (four days to two weeks) and bile duct decompression on metabolic parameters in the rat.

### MATERIALS AND METHODS

The experiments were performed on 160 male Fischer-344 rats (body weight: 275–300g; Charles River Breeding Laboratories, Inc.). The animals were allowed Purina Chow and water ad libitum. They were placed in individual cages five days prior to the start of the experiment for environmental adaptation.

The rats were randomly separated into eight groups (10 rats in each group), according to the duration of jaundice and decompression. All these groups had equal numbers of pair-fed control rats, submitted to similar sham operative procedures.

The experimental groups were:

— 4D (sacrificed after being jaundiced for four days).
— 4D1W (jaundiced for four days, then decompressed. Sacrificed one week later).
— 1W (sacrificed after being jaundiced for one week)
— 1W1W (jaundiced for one week, then decompressed. Sacrificed one week later).
— 1W2W (jaundiced for one week, then decompressed. Sacrificed two weeks later).
— 2W (Sacrificed after being jaundiced for two weeks).
— 2W1W (jaundiced for two weeks, then decompressed. Sacrificed one week later).
— 2W2W (jaundiced for two weeks, then decompressed. Sacrificed two weeks later).

### *Bile Duct Ligation*

For this experiment, we used the model for reversible obstructive jaundice developed in our laboratory[4]. Briefly, all surgical procedures were performed under aseptic conditions. The rats were anesthetized with IP pentobarbital (50 mg/kg). After laparotomy, the common bile duct was identified and dissected free from adjacent organs. A vessel loop was then passed behind the duct, with great care to avoid damage to the hepatic artery or portal vein. The loop was exteriorized through the abdominal wall and fixed to the muscular layer under the skin, applying tension on the vessel loop. The abdominal wall was closed with continuous 3-0 silk suture in two layers: muscular and cutaneous. The rats were then returned to their individual cages.

To reverse the bile duct obstruction, the rats were anesthetized with IP pentobarbital (50 mg/kg), and a small incision (3-4 mm) was performed over the site of fixation of the vessel loop. The sutures that hold the vessel loop in place were cut and the vessel loop pulled out gently. The skin was then closed with one stitch of 3-0 silk, and the rats returned to their cages.

The control group was submitted to similar procedures, except for the passage of the vessel loop behind the bile duct. These rats were pair fed to the food intake of their corresponding pair. All the rats had their body weight and food intake determined daily.

On the day of sacrifice, the rats were anesthetized with IP pentobarbital (50 mg/kg), a blood sample for biochemical determinations was drawn from the abdominal aorta. Inspection of the liver and bile ducts was systematically performed. The biochemical determinations in the blood samples were: Free fatty acids (FFA)-(Wako Pure Chemical Ind. Ltd), and triglycerides (TG), glucose (GLU), total bilirubin (BILI), albumin (ALB), and liver enzymes (Alanine Amino Transferase-ALT: EC 2.6.1.2; Aspartate Amino Transferase-AST: EC 2.6.1.1; Alkaline Phosphatase-AP: EC 3.1.3.1) determined in a Technicon RA-500, Technicon Instruments Corp.). The care of all our laboratory animals is in compliance with the Guide for the Care and Use of Laboratory Animals. We are also in compliance with Animal Welfare Act and adhere to the Public Health Service "Principles for the Use of Animals" (NIH Manual, chap. 4206).

Statistical analysis: The results are reported as mean +/− standard error of the mean (SEM). In each group, values from test animals and their pair-fed controls were compared using ANOVA (analysis of variance). Single asterisk, $P<0.05$, Double asterisk, $P<0.02$. The null hypothesis was rejected at $=0.05$. Mortality was analyzed according to the method of Kaplan and Meier[5].

### RESULTS

Following bile duct obstruction, we observed a decrease in food intake and body weight in all rats. This decrease reached a minimum level around postoperative day 4, stabilizing thereafter. There was a parallel decrease in body weight in the pair-fed control animals, and there was no significant difference between the groups and their controls. Following decompression, there was a progressive increase in the body weight of the animals, which was maintained until the day of sacrifice. There was no significant difference in body weight between jaundiced and sham operated, pair-fed control animals (Figure 1).

Mortality was not significantly different between sham operated controls and the rats submitted to jaundice for periods of four days or one week ($P=0.7832$ and $P=0.6625$, respectively). The jaundiced state maintained for two weeks, however, significantly affected survival of the animals, with most of the deaths occurring after 9–10 days of bile duct obstruction ($P=0.00007$). On autopsy, we observed profuse intra-abdominal hemorrhage in all these rats.

Reversal of bile duct ligation was possible in all the rats submitted to the procedure for four days, but 3% were not reversed by the removal of the loop after one week of obstruction. This proportion increased to 10% in the group of animals jaundiced for two weeks.

Bilirubin: Bile duct obstruction as expected, significantly increased bilirubin levels (Figure 2). However, there was a trend towards a progressive decrease of bilirubin levels with more prolonged jaundice (4D, 1W, and 2W). Following decompression, there was a significant drop in bilirubin levels, reaching normal range in all groups after one week, although still higher than pair-fed controls. After two weeks, bilirubin levels were similar to those recorded in the pair-fed controls.

### *Metabolic Profile*

Free Fatty Acids: Figure 3 shows that following bile duct obstruction (4D, 1W, and

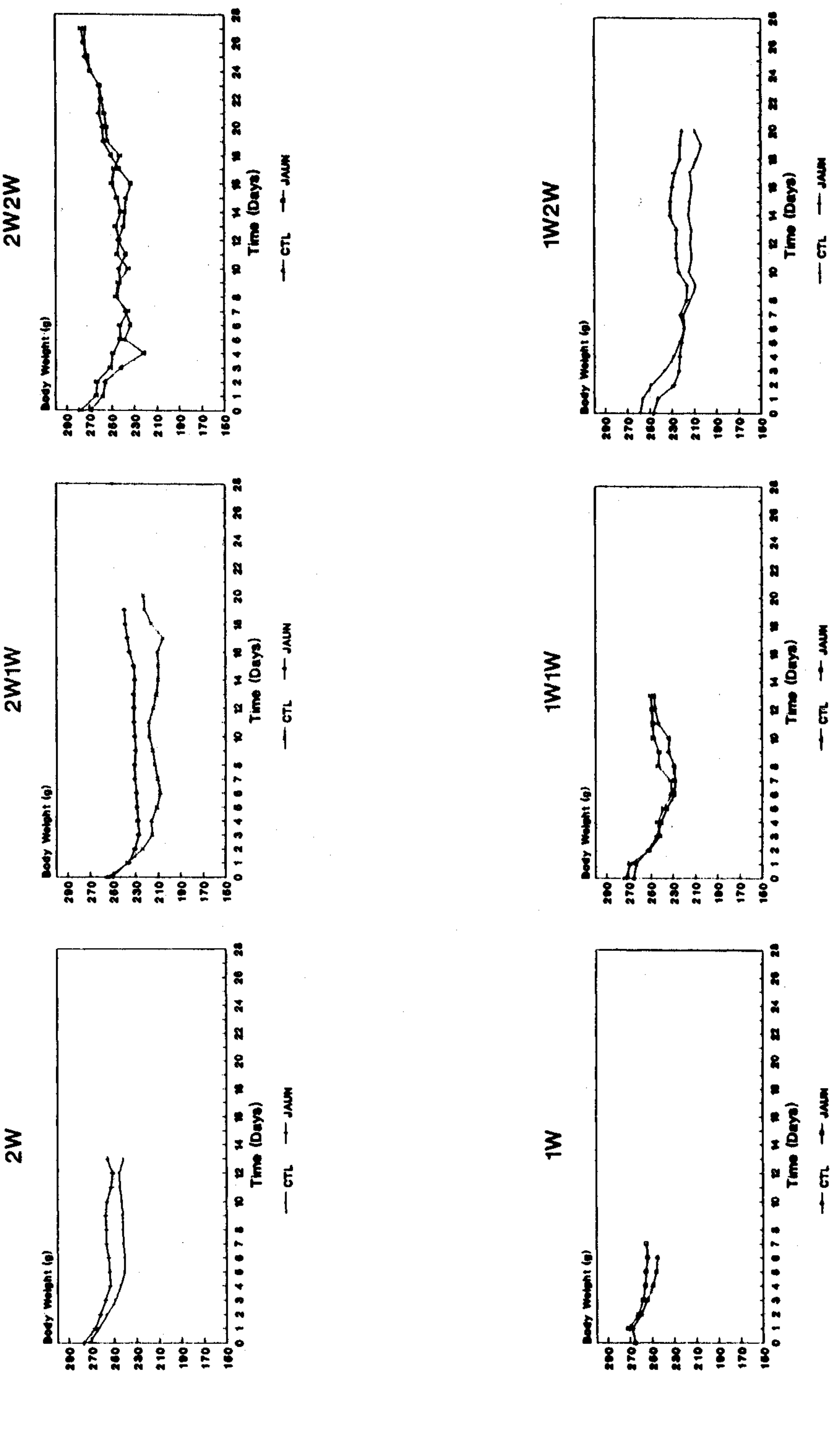

Figure 1

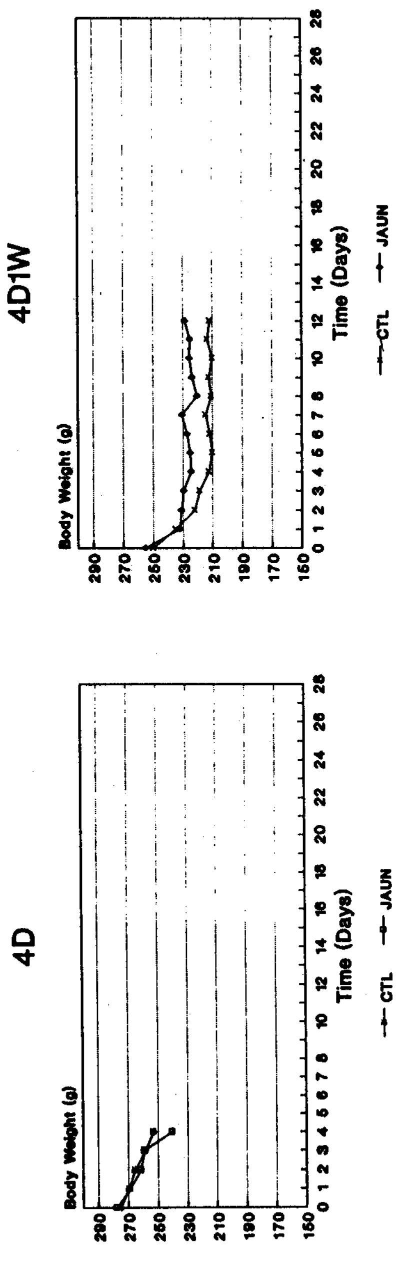

Figure 1 (cont)

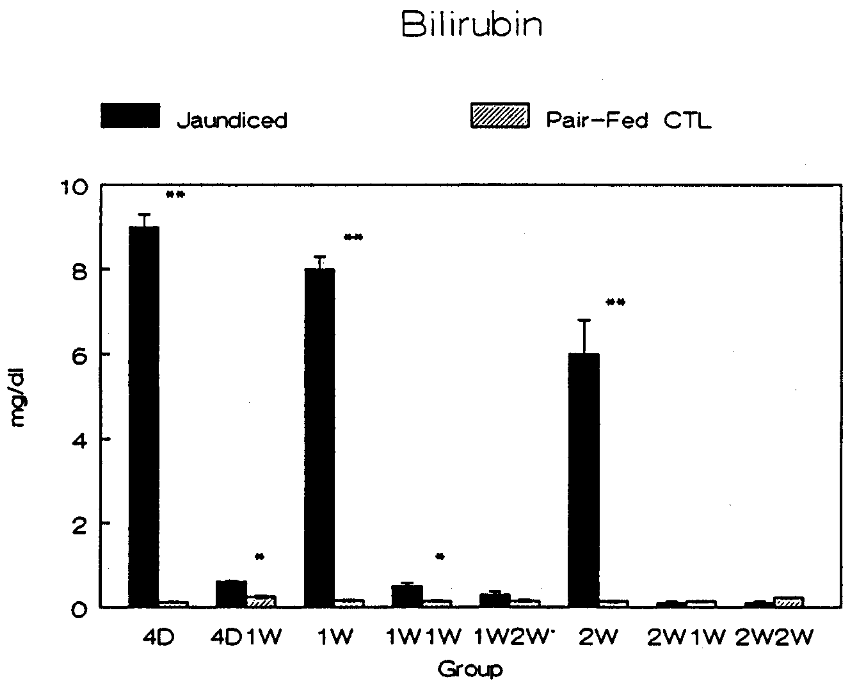

**Figure 2**

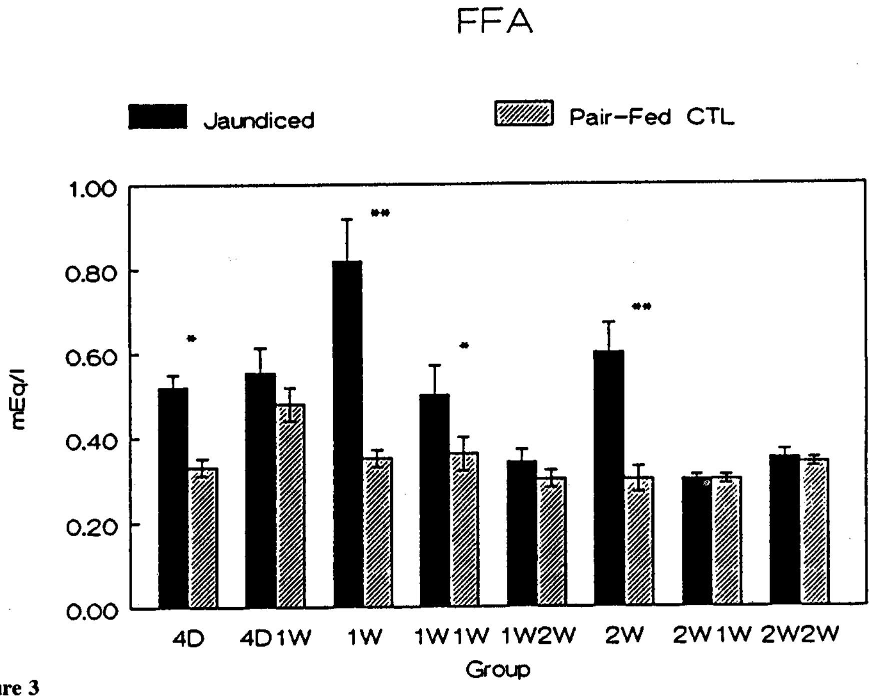

**Figure 3**

2W), FFA increased significantly. FFA returned to control levels after decompression in all groups after one week (except for 1W1W). After two weeks, all animals had levels of FFA within the control range.

Triglycerides: Following bile duct obstruction, TG levels increased significantly, compared to sham operated pair-fed controls (4D, 1W and 2W). However, as shown in Figure 4, decompression of bile duct for one week was not sufficient to totally reverse hypertriglyceridemia, although there was a clear decrease in TG levels and a trend towards normalization. In the rats that were jaundiced for two weeks and decompressed for two other weeks, TG levels returned to control levels.

Glucose: Bile duct obstruction affected glucose levels significantly even after 4 days of jaundice (Figure 5). Jaundiced rats were consistently hypoglycemic when compared to normal pair-fed controls (4D, 1W, and 2W). After one week of decompression, glucose levels in the jaundiced rats increased significantly, reaching control levels, and in one instance (4D1W), glucose levels were higher than control animals.

Albumin: Two weeks of bile duct obstruction were necessary in order to significantly decrease albumin levels, compared to sham-operated controls. Albumin levels did not return to normality in these animals, even following two weeks of decompression (Figure 6).

### *Liver Function Tests*

Alanine Amino Transferase: Figure 7 shows that bile duct obstruction increased significantly ALT levels, with the highest increase observed after 4 days of obstruction. Relief of obstruction tended to decrease ALT levels, but normal levels were not attained during the 2 weeks following decompression (except for group 1W1W).

Aspartate Amino Transferase: Figure 8 shows that bile duct obstruction promoted a significant increase in the AST levels. With prolonged jaundice, however, AST levels tended to decrease progressively. In spite of a decrease in AST concentration following decompression, the level of this enzyme did not reach normal levels during 2 weeks following decompression.

Alkaline Phosphatase: Alkaline phosphatase levels increased steadily following bile duct obstruction (Figure 9). Decompression of bile duct decreased AP levels, and after two weeks all rats had comparable normal levels.

### DISCUSSION

In the present study, the effect of extrahepatic bile duct obstruction prolonged over increasing periods of time (from four days to two weeks) and the effect of biliary decompression on various liver enzymes as well as metabolic function variables were evaluated. We extended our earlier studies to evaluate the reproducibility and feasibility of a reversible jaundice model in rats developed in our laboratory, but used in this study for more prolonged periods of time. This model of reversible obstructive jaundice is feasible, with few inherent complications. The efficiency of reversal of bile duct obstruction by this method is not absolute, and with prolonged

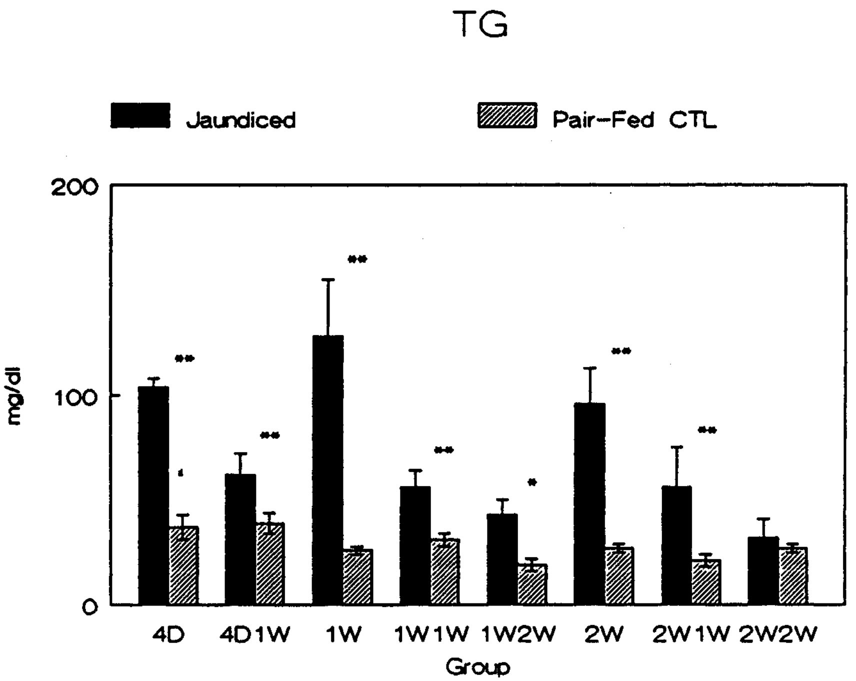

**Figure 4**

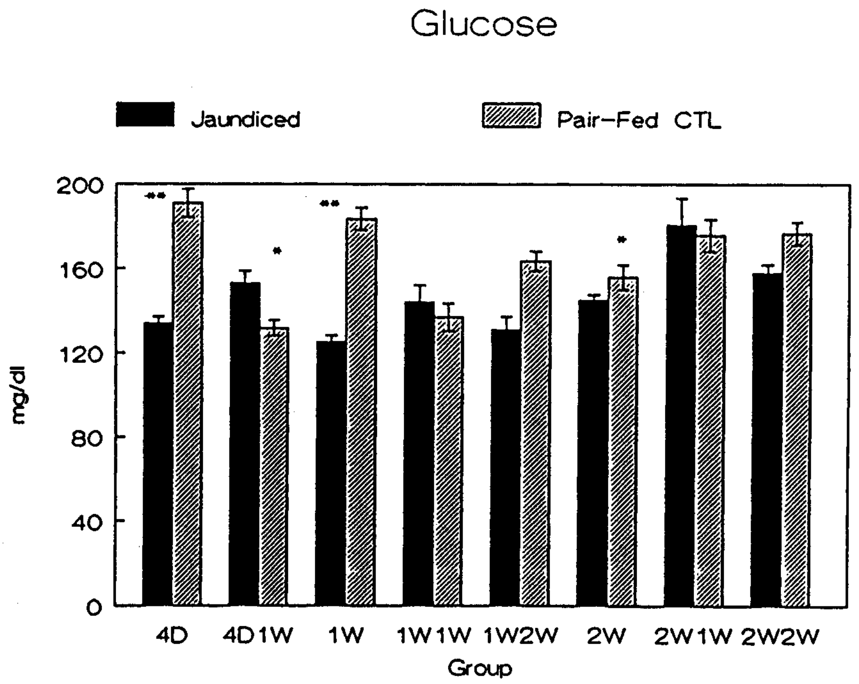

**Figure 5**

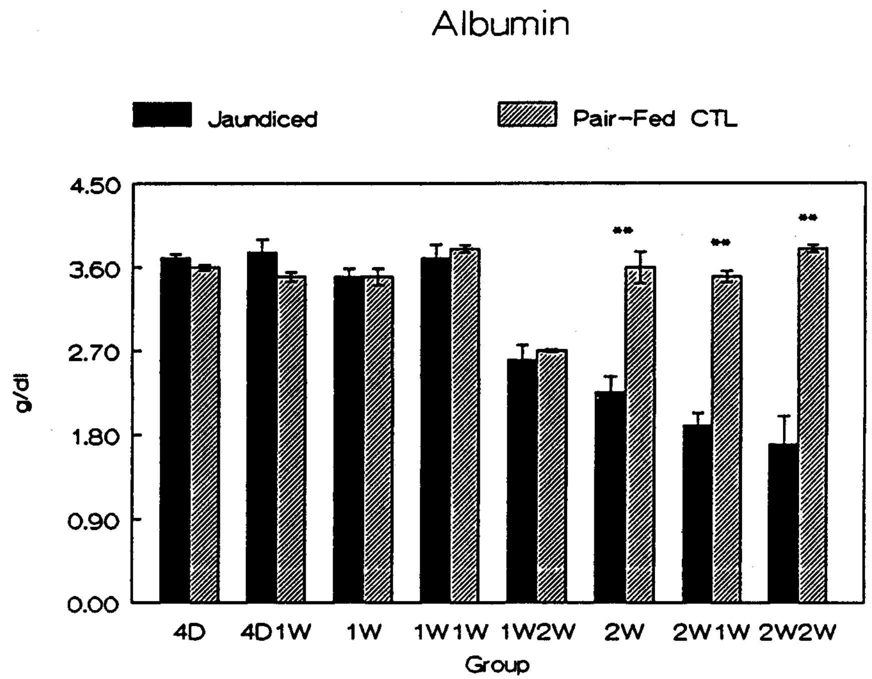

Figure 6

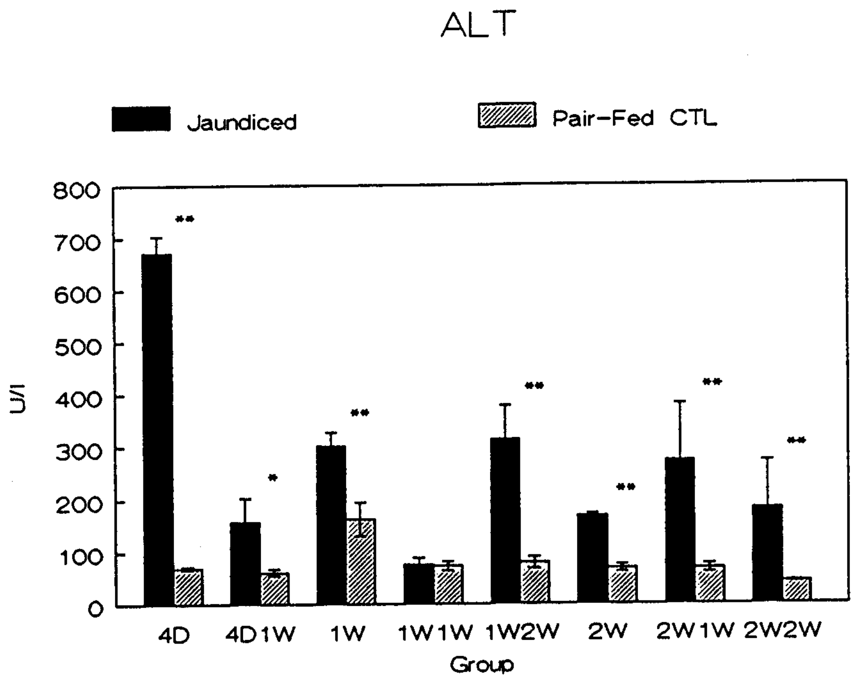

Figure 7

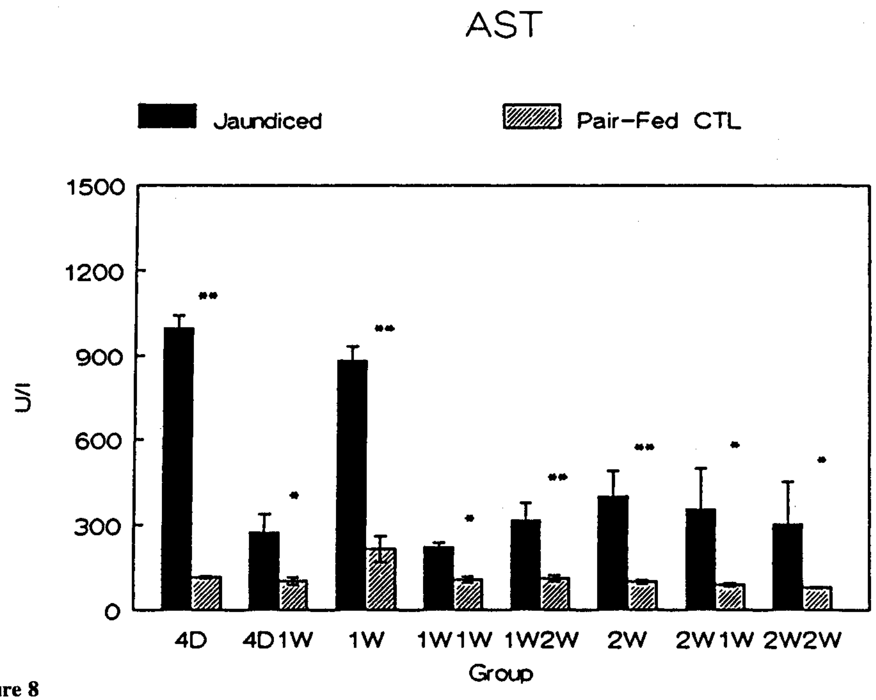

**Figure 8**

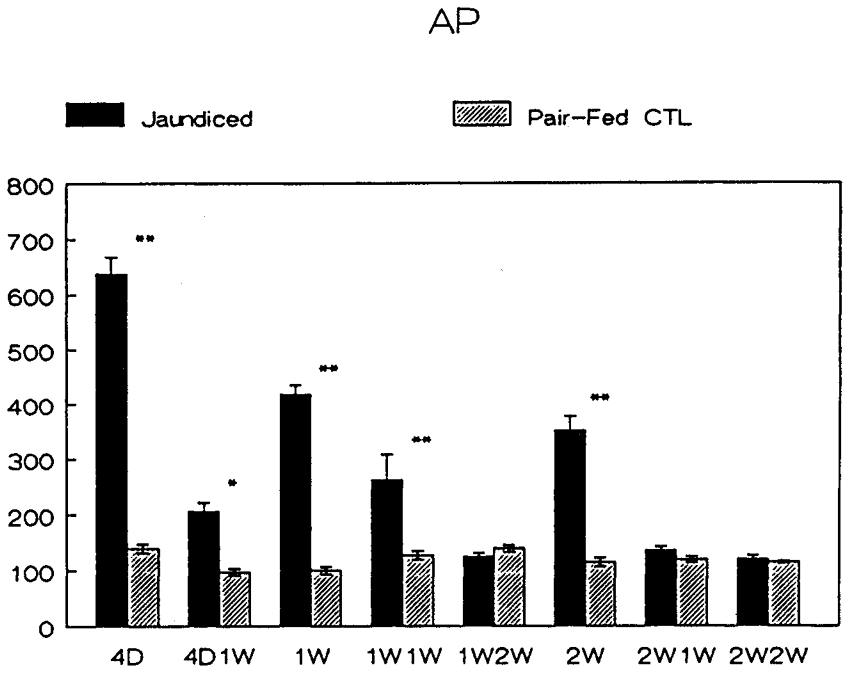

**Figure 9**

obstruction, increasing numbers of rats are likely to remain jaundiced after the removal of the obstructing loop, mostly because of adhesions that maintain the bile ducts in an angulated position that prevents free passage of bile. In the original description of the reversible jaundice model[4], the authors observed the occurrence of peritonitis in the postoperative period. We modified their model slightly in order to avoid further contamination of the peritoneal cavity by suturing the loop that obstructs the bile duct to the muscular layer of the abdominal wall, then closing completely the skin overlying the exteriorized vessel loop. In this fashion the loop was isolated from the external milieu, and would not be a vehicle for the entry of microorganisms into the peritoneum. Prolonged jaundice (more than 10 days) was associated with an increased mortality mainly from intra-abdominal hemorrhage, which attests to a marked decrease in the blood coagulation. This might result from a decrease in the capacity of the liver to synthesize blood clotting factors, but these were not measured. The plasma albumin levels suggest that the synthetic function of the hepatocytes could be decreased for albumin production, with significant hypoalbuminemia observed following two weeks of jaundice. Progressive hypoalbuminemeia was also reported by other investigators[6]. This alteration was not reversed by bile duct decompression, at least not for 14 days post-decompression. One week or less of jaundice did not alter albuminemia significantly, compared to sham operated, pair-fed controls. More than one week of jaundice is required before the albumin concentration falls, and the failure of reversal of this concentration after decompression suggest impaired synthesis. This observation is consistent with a half-life for albumin of approximately 10 days. Liver function tests rapidly increased with biliary obstruction, and gradually returned towards normal levels after decompression, along with bilirubin levels. The dysfunction of glucoeogenesis, reflected by relative hypoglycemia, was also reversed following bile duct de-obstruction. Gluconeogenesis was markedly decreased in rats with bile duct ligation, even when substrate for the reaction was offered with no restriction[7]. In obstructive jaundice, plasma levels of glucogenic amino-acids and lactate and glycerol, the principal substrates of gluconeogenesis are present at normal or elevated levels[8]. Alteration of activity of enzymes involved in glucose production are described in association with jaundice. Glycogen phosphorylase activity was decreased in hepatocytes isolated from cholestatic animals, along with a modification of the balance between alpha1 and beta2 adrenergic receptors in the liver, with a shift in the control of the stimulation of glycogen phosphorylation[9]. Lipid metabolism was significantly affected by jaundice, with the increase in circulating FFA and TG levels. Hyperlipemia has been observed previously in association with obstructive jaundice[8,10,11,12]. The observed hyperlipemia could result from different mechanisms: plasma FFA increase could result from accelerated lipolysis, with or without decrease in the peripheral uptake of FFA. Hypertriglyceridemia could result from an increased release by the liver, or a decreased clearance of the circulating TG by the peripheral tissues. The exact mechanisms underlying our observations remain to be elucidated.

The results of this study show that decompression of the bile duct could reverse most of the biochemical alterations observed in the jaundiced animal. The *timing* of the *decompressive* procedure appears to be crucial if the purpose is to reverse, and presumably preserve, the synthetic function of the hepatocytes. In our model, jaundice prolonged for two weeks decreased markedly plasma albumin levels, and decompression did not reverse this alteration, even though liver function tests and bilirubin levels returned to normal levels. These results suggest that reliance on bilirubin levels, or other function tests, to decide on the timing of relief of bile duct obstruction, or to evaluate the efficacy and the "benefits" of decompression, is not totally accurate. The significance of standard liver function tests was found to be of limited value in our study, and in other reported studies. The recovery of the liver function to within the normal range is somewhat rapid after release of obstruction[6], even though basic metabolic pathways (e.g., mitochondrial respiration) are still impaired. More accurate tests should be sought to evaluate liver function reserve, and functional recovery following any therapeutic procedures. According to our study, when decreased albumin levels were observed, decompression did not reestablish synthetic liver function. This is in agreement with earlier findings that decrease in protein and albumin content was also observed with little recovery after relief of obstruction[6]. Therefore waiting for albumin levels to decline might delay early intervention. In contrast, the earlier the obstruction is relieved the higher is the likelihood of reversing the metabolic alterations associated with jaundice. In recent years, instead of operative relief of biliary obstruction, nonsurgical percutaneous biliary drainage has been examined[2], followed by radical operation. The results failed to show significant impact on morbidity and mortality of the patients. The experimental data demonstrate that, in order to perform this radical operation safely, the interval between external biliary drainage and operation should be as long as possible during which time the improvement of mitochondrial function could be obtained (4–6 weeks after drainage)[6]. Further studies would elucidate the best timing for decompression and for any further surgical treatment.

We conclude that the reversible jaundice model is feasible, with few limitations if used for jaundice periods longer than a week. Prolonged jaundice has a profound effect on the intermediary metabolism, with alterations in lipid, carbohydrate and protein metabolism. Biliary decompression reversed effectively most of these alterations following 4 day or one week jaundice, but was unable to reverse hypoalbuminemia after two weeks jaundice. Further studies should elucidate the mechanisms underlying the metabolic alterations to further clarify the pathophysiology of obstructive cholestasis.

*Acknowledgements*

We thank Charles C. Ahrens and Bruce H. Ng for their excellent technical assistance.

*References*

1. Norlander, A., Kalin, B. and Sunblad, R. (1982) Effect of percutaneous transhepatic drainage upon liver function and postoperative mortality. *Surg. Gynecol. Obstet.*, **155**, 161–166
2. McPherson, G.A.D., Benjamin, I.S., Hodgson, H.J.F., Bowley, N.B., Allison, D.J. and Blumgart, L.H. (1984) Pre-operative percutaneous transhepatic biliary drainage: the results of a controlled study. *Br. J. Surg.*, **71**, 371–375
3. Hardison, W.C., Weiner, R.G., Hatoff, D.E. and Miyai, K. (1983) Similarities and differences between models of extrahepatic biliary obstruction and complete biliary retention without obstruction in the rat. *Hepatology*, **3**, 383–390
4. Posner, M.C., Burt, M.E., Stone, M.D., Han, B.L., Warren, R.S., Vydelingum, N.A. and Brennan, M.F. (1990) A model of reversible obstructive jaundice in the rat. *J. Surg.Res.*, **48**, 204–210
5. Kaplan, E.L. and Meier, P. (1958) Nonparametric estimation from incomplete observations. *Am. Stat. Assoc. J.*, **53**, 457–481

6. Koyama, K., Takagi, Y., Ito, K. and Sato, T. (1981) Experimental and clinical studies on the effect of biliary drainage in obstructive jaundice. *Am. J. Surg.*, **142**, 293–299
7. Lee, E. and Haines, J.R. (1972) The effect of experimental bile-duct obstruction on critical biosynthetic functions of the liver.*Brit. J. Surg.*, **59**, 564–568
8. Record, C.O., Alberti, K.G. and Williamson, D.H. (1972) Lipid metabolism in experimental liver disease resulting from (D( + )-galactosamine administration. *Biochem. J.*, **130**, 37–44
9. Aggerbeck, M., Ferry, N., Zafrani, E.S., Billon, M.C., Barouki, R. and Hanoune, J. (1983) Adrenergic regulation of glycogenolysis in rat liver after cholestasis. *J. Clin. Invest.*, **71**, 476–486
10. Center, S.A., Baldwin, B.H., King, J.M. and Tennant, B.C. (1983) Hematologic and biochemical abnormalities associated with induced extrahepatic bile duct obstruction in the cat. *Am. J. Vet. Res.*, **44**, 1822–1829
11. Mortiaux, A., and Dawson, A.M. (1961) Plasma free fatty acid in liver disease. *Gut*, **2**, 304–309
12. Starnes, H.F., Conti, P.S., Warren, R.S., Jeevanandam, M. and Brennan, M.F. (1987) Altered peripheral amino acid uptake in obstructive jaundice. *J. Surg. Res.*, **42**, 383–393

*(Accepted by S. Bengmark 15 March 1991)*

*Footnote*

This study was supported by AM-34141 (NAV) and the Surgical Metabolism Fund

### INVITED COMMENTARY

Studies on the changes occurring following biliary obstruction as well as the normalization after biliary decompression are of utmost importance as the value of preoperative biliary drainage is still debated. Thus, the exact pathophysiological mechanisms of the morphological and functional changes in the liver, including hepatocyte and Kupffer cell function, are not fully determined. Furthermore, the intervals for the restoration of these functions are not known, whereby the optimal length of preoperative biliary drainage is not clear. In the present paper, the authors present some variables reflecting liver and metabolic functions following biliary obstruction and biliary decompression using an elegant model for reversible obstructive jaundice in the rat[1]. In order to obtain a model of "chronic biliary stasis", two weeks of biliary obstruction is usually required in the rat, which also is shown in this paper. Standard liver function enzymes more or less normalized within a period of two weeks of biliary decompression in the jaundiced rats. This normalization, however, does not correlate to the restoration of hepatocyte mitochondrial function, Kupffer cell reticuloendothelial function and the morphological changes induced by "chronic" biliary obstruction, which require a longer period in order to normalize[2,3]. A remaining hepatocyte secretory dysfunction is also indicated by the persisting low albumin levels following two weeks of biliary decompression in the present study. Thus, it would be interesting to evaluate longer periods of biliary decompression, including other parameters of mitochondrial function, e.g. antipyrine clearance[4] and reticuloendothelial system function. As surgery in the jaundiced patient is associated with high morbidity, not least infectious complications[5], evaluation of the influence of biliary obstruction and biliary decompression on mortality could preferably be performed by studying the outcome of a septic challenge. Further studies in this field are necessary in order to clarify the pathophysiological mechanisms of the restoration of hepatic function following biliary obstruction and biliary decompression and the optimal timing of surgery.

### *References*

1. Posner, M.C., Burt, M.E.,Stone, M.D., Hahn, B.L., Warren, R.S., Vydelingum, N.A. and Brennan, M.F. (1990) A model of reversible obstructive jaundice in the rat. *J. Surg. Res.*, **88**, 204–210
2. Koyama, K., Takagi, Y., Ito, K., and Sato, T. (1981) Experimental and clinical studies on the effect of biliary drainage in obstructive jaundice. *Am. J. Surg.*, **142**, 293–299
3. Ryan, C.J., Than, T., Blumgart, L.H. (1977) Choledochoduodenostomy in rats with obstructive jaundice. *J. Surg. Res.*, **23**, 321–331
4. McPherson, G.A.D., Benjamin, I.S., Boobis, A.R. and Blumgart, L.H. (1985) Antipyrine elimination in patients with obstructive jaundice: a predict of outcome. *Am. J. Surg.*, **149**, 140–142
5. Gundri, S.R., Strodel, W.E., Knol, J.A., Eckhauser, F.D. and Thompson, N.W. (1984) Efficacy of preoperative biliary tract decompression in patients with obstructive jaundice. *Arch. Surg.*, **119**, 703–707

Roland Andersson
Department of Surgery
Lund University
S-221 85 Lund, Sweden

